# A conserved phosphorylation mechanism for regulating the interaction between the CMG replicative helicase and its forked DNA substrate

**DOI:** 10.1016/j.jbc.2025.108408

**Published:** 2025-03-14

**Authors:** Sandra Koit, Nele Tamberg, Allan Reinapae, Lauri Peil, Arnold Kristjuhan, Ivar Ilves

**Affiliations:** 1 Institute of Technology, University of Tartu, Tartu, Estonia; 2 Institute of Molecular and Cell Biology, University of Tartu, Tartu, Estonia

**Keywords:** eukaryote, DNA replication, DNA helicase, checkpoint control, protein phosphorylation

## Abstract

The CMG helicase is a crucial enzyme complex that plays a vital role in the replication of genomic DNA in eukaryotes. Besides unwinding the DNA template and coordinating the replisome’s structure, it is also a key target for signaling pathways that regulate the replication process. We show that a specific serine/threonine residue in the MCM3 subunit of CMG, which has been previously linked to phosphorylation-dependent control mechanisms of genomic DNA replication in human cells, is a conserved phosphorylation site for Chk1 and potentially other protein kinases. This suggests a conserved regulatory mechanism associated with it in metazoans and several other eukaryotes, including budding yeast. Our *in vitro* analysis links this mechanism directly to the modulation of the CMG helicase activity by impacting its interactions with the forked DNA substrate. Further supporting its conserved role in regulation, we found that phosphomimetic substitution with aspartic acid and alanine knockout of this conserved residue lead to opposite phenotypic defects in the growth of budding yeast cells. These findings outline a candidate conserved phosphorylation pathway for regulating genomic DNA replication in eukaryotes, which adjusts the interactions between the replicative helicase complex and its DNA substrate according to the specific needs of various physiological conditions.

DNA replication is a fundamental biochemical process essential for maintaining genome integrity. In eukaryotes, the overall mechanism of genomic DNA replication is conserved from yeast to humans. However, the exact details and participating factors vary among organisms, particularly regarding regulation. This helps to ensure optimal synchrony within the replication mechanism and coordination with other cellular processes in different organismal contexts and environmental conditions.

One of the crucial and well-conserved enzyme complexes in genomic DNA replication is the eleven-subunit Cdc45-MCM2-7-GINS (CMG) helicase, which prepares template DNA for the synthesis of complementary strands by unwinding it in front of the replisome (1, 2, 3, 4). The CMG helicase is also central in coordinating the replication initiation process. The loading of the dormant heterohexameric MCM2-7 motor part of CMG on DNA determines the potential replication start sites in the genome. The activation of CMG helicase by the assisted recruitment of Cdc45 and a four-member GINS complex to MCM2-7 triggers replisome assembly and determines the actual start sites and timing of replication initiation (5). Within the progressing replisome, CMG provides structural coordination of the unwinding template with the leading and lagging strand replication machinery (6, 7). Finally, at the completion of DNA synthesis, assisted disassembly of CMG contributes to the correct termination of genome replication (8, 9). All of these processes are strictly directed and overseen by a complex network of molecular control pathways, altogether ensuring the precise, timely, and complete duplication of genomic DNA before each cell division.

Protein kinases play a prominent role in the regulation of genomic DNA replication in eukaryotes by phosphorylating and modulating the activity and interactions of key components of the replisome and associated factors, including CMG helicase. The critical roles of the cell cycle kinases cyclin-dependent kinase and Dbf4-Cdc7 (DDK) in controlling CMG helicase assembly and activation during the initiation of genomic DNA replication are well documented in budding yeast. DDK facilitates this process by phosphorylating the disordered regulatory tails of MCM4 and MCM6 subunits (10, 11, 12), whereas cyclin-dependent kinase phosphorylates Sld2 and Sld3 proteins, which together assist in the recruitment of GINS and Cdc45 auxiliary subunits of CMG to the MCM2-7 motor complex (13, 14, 15). Both kinases are also involved in the regulation of replication initiation in Metazoa. However, the assembly of CMG has evolved into a more complex process in this phylogenetic group, and many of its details, including the participating protein factors, have diverged significantly from those used in budding yeast (16, 17, 18, 19, 20, 21).

Another important group of regulatory factors consists of cell cycle checkpoint kinases that control genome replication in response to potentially genotoxic stress conditions. In budding yeast, these conditions trigger the activation of Mec1 and its downstream Rad53 checkpoint kinases. To provide the time for damage containment, Rad53 then blocks the initiation from late replication origins by phosphorylating and inactivating the CMG assembly factor Sld3 and the Dbf4 regulatory subunit of DDK kinase (22, 23). Mec1 and Rad53 can control genomic replication also through several additional mechanisms, most notably by directly regulating dNTP production, slowing down replication forks, and protecting stalled replication forks for subsequent restart (24, 25). Similarly, in Metazoa, ATR and its downstream Chk1 checkpoint kinases are essential in controlling genome replication through several mechanisms (26, 27). In response to genotoxic replication stress conditions, Chk1 inhibits DNA synthesis by stopping cell cycle progression through phosphorylation-dependent degradation of Cdc25A phosphatase (28, 29) and by directly regulating late origin firing (30, 31). Like the Mec1–Rad53 pathway in yeast, the metazoan ATR–Chk1 pathway has been linked to the mechanisms that maintain the stability of stalled replication forks (32). In addition, the regulation of DNA replication by checkpoint kinases is not limited to stress response. In the normal unperturbed S-phase, the ATR–Chk1 pathway controls the overall rate of DNA replication by regulating the frequency of origin firing (33).

The CMG helicase complex, which moves in front of the replisome and coordinates it structurally, is an attractive target for regulating replication forks’ stability and progression. Several regulatory kinases have been shown to phosphorylate CMG subunits, and some evidence suggests that specific modification pathways can indeed directly target the activity of this critical component of the replisome (34, 35). One example is the phosphorylation of a specific residue in the MCM3 subunit of human CMG by Chk1, both *in vitro* and *in vivo*, which has been linked to the phenotypic effects related to genomic DNA replication (36). Here, we present evidence that this Chk1-dependent regulation mechanism directly targets the activity of the CMG replicative helicase by modulating its interactions with the forked DNA substrate. Our analysis suggests the evolutionary conservation of the described phosphorylation-dependent regulatory mechanism in metazoans and perhaps some earlier diverged phylogeny groups.

## Results

### Chk1 kinases from fruit flies, humans, and mice phosphorylate a conserved target residue in an MCM3 subunit of the CMG replicative helicase complex

**Figure 1:**
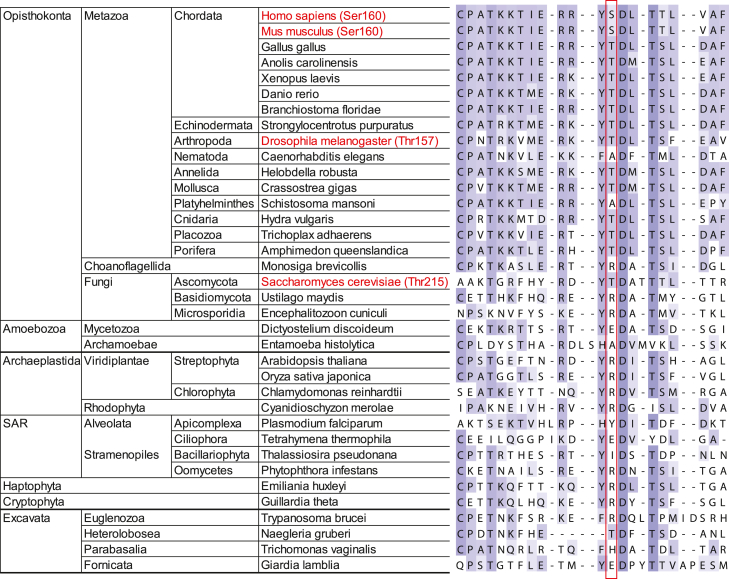
**Sequence alignment of the subregion in eukaryotic MCM3 proteins containing the characterized conserved S/T position (marked with a *red* box across the sequences).** The species list was compiled to sample all the main groups of the consensus eukaryotic phylogenetic tree (72), with a more detailed emphasis on Metazoa. The relative conservation of the residues is presented by *violet* shading (a *darker color* corresponds to better conservation). Specific positions in the MCM3 proteins characterized in this study and the respective species are marked in *red*.

In the human MCM3 protein, a single amino acid in the N-terminal domain of the protein (S160 or S205, depending on annotation) has been identified as a significant phosphorylation target site of the Chk1 checkpoint kinase (36). Our homology alignment of MCM3 proteins from representative species across the eukaryotic phylogenetic tree revealed that the serine/threonine residue in this position is conserved in metazoans and some earlier diverged phylogenetic groups (Fig. 1). In most of the Metazoan MCM3 sequences, this residue is located in the context of previously characterized human Chk1 consensus site [M/I/L/V]-X-[K/R]-X-X-[S/T] (37). The surrounding sequence beyond the region shown in the figure also aligns well and exhibits good homology in all eukaryote MCM3 proteins. These features are uncommon for regulatory phosphosites in MCM2-7, which primarily cluster into nonstructured tails and internal regions of the subunit proteins with limited sequence conservation. This suggested about the potential involvement of the characterized position in a conserved phosphorylation-dependent regulation mechanism in eukaryotes. However, in an earlier study, a Chk1 kinase from the fruit fly *Drosophila melanogaster* could not phosphorylate MCM2-7 or other subunits of the CMG complex. Closely related Chk2 checkpoint kinase from the same organism phosphorylated MCM3 and two other CMG subunits. However, its target residues were determined not to include the T157 position of the fruit fly MCM3 protein, which corresponds to the Chk1 target residue S160 in human MCM3 (35). These data seemingly contradicted the possibility of a conserved phosphorylation mechanism associated with the mentioned residue.

To address this question, we purified baculovirus-expressed Chk1, Chk2, and MCM2-7 proteins from *Drosophila* (Dm), humans (Hs), and mice (Mm), as well as complete CMG complexes from mice and *Drosophila*. Baculovirus expression has been previously shown to yield active human Chk1 and Chk2 protein kinases (37, 38, 39, 40), and all our recombinant Chk1 and Chk2 proteins indeed efficiently phosphorylated Cdc25-derived Chk1 and Chk2 substrate peptide commonly used for testing the activity of Chk kinases (Fig. S1, *A*–*C*). The histone H1 protein, which has been previously used as a test substrate for a closely related yeast Rad53 checkpoint kinase (41), was also found to serve as a good test substrate for metazoan Chk kinases and was phosphorylated by all purified Chk1 and Chk2 kinases (Fig. S1*D*).

**Figure 2:**
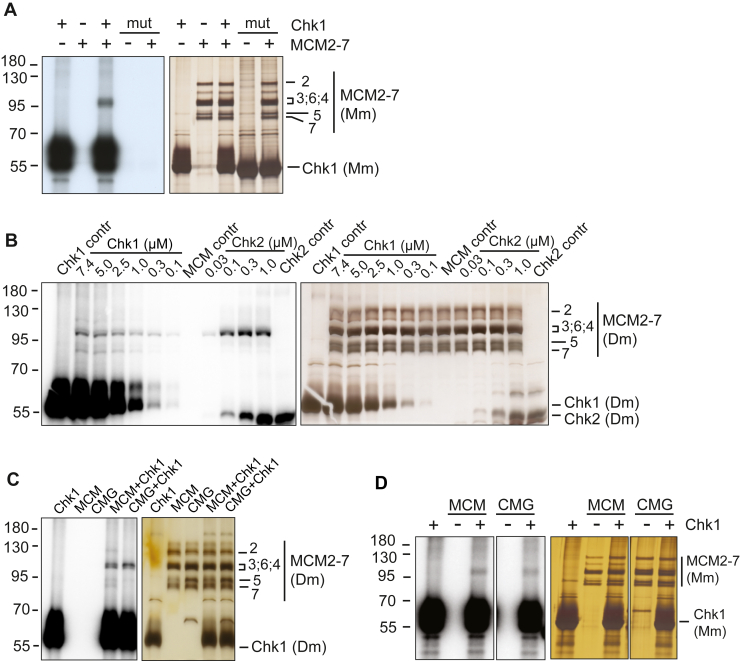
**Kinase assays showing phosphorylation of Drosophila (Dm) and mouse (Mm) MCM2-7 and CMG complexes by Chk1 and Chk2.** Silver-stained 10% SDS-PAGE protein gels are shown on the *right*, and X-ray film (*A*) or PhosphoImager scan images (*B*–*D*) of the same gels are shown on the *left*. Here and in other kinase assay images, the numbers on the *left* correspond to the mobility of the molecular weight markers (kD). *A*, kinase assay using mouse Chk1 and MCM2-7 proteins (4.1 μM Chk1 and 50 nM MCM2-7). “mut” corresponds to the lanes containing reactions with kinase-defective mutant of Chk1. *B*, comparative kinase titration assay with *Drosophila* Chk1 and Chk2 kinases, using 100 nM MCM2-7 as a substrate. The kinase concentrations are shown at the *top* of each lane. “contr” marks the control reactions with the proteins shown alone. The quantified signal from the MCM3 region of the PhosphorImager scan images is shown on the Fig. S1. *C*, comparison of *Drosophila* Chk1-dependent (2 μM) phosphorylation of MCM2-7 (40 nM) and CMG (40 nM). *D*, comparison of the mouse Chk1-dependent (2.3 μM) phosphorylation of MCM2-7 (50 nM) and CMG (10 nM). Lanes were cut from the same autoradiographs and protein gel images.

Next, we used these recombinant proteins to carry out side-by-side *in vitro* protein kinase assays with MCM2-7 substrates, testing Chk1 kinases first. These experiments revealed that MCM2-7 is phosphorylated by Chk1 not only in humans (36) but also in mice (Fig. 2*A*) and *Drosophila* (Fig. 2*B*). However, roughly ten times higher concentrations of Chk1 than *Drosophila* Chk2 were required to reach the same levels of MCM2-7 phosphorylation (Fig. 2*B*, and S1, *E* and *F*). This difference was not due to the overall lower activity of recombinant Chk1 proteins, as all three purified Chk1 kinases phosphorylated the Cdc25 and histone H1 test substrates with similar or better activity than *Drosophila* Chk2 (Fig. S1). The inactivating D>N substitution of the catalytic residue abolished the Chk1 kinase autophosphorylation and the phosphorylation of MCM2-7 subunits (Figs. 2*A* and S2*A*). Therefore, both the kinase autophosphorylation and MCM2-7 phosphorylation signal are due to the intrinsic activity of the recombinant Chk1 kinase. This confirmed the Chk1-specific phosphorylation in our assays' relatively high kinase-to-substrate ratios, which we had to use due to the low concentrations of our recombinant MCM2-7 and CMG proteins. While the apparent affinity of the Chk1 kinase to MCM2-7 is clearly lower than in the case of *Drosophila* Chk2, the phosphorylation itself is rather effective on the saturating levels. We estimated that in these assays, *Drosophila* Chk1 phosphorylated, on average, close to one residue per MCM2-7 complex at saturating (low micromolar) kinase concentrations. This estimation is based on observations that in the kinase titration assays, the MCM2-7 phosphorylation signal with *Drosophila* Chk1 plateaued at a level that was roughly half of the saturation signal in the case of *Drosophila* Chk2 (Figs. 2*B* and S1, *E* and *F*), and Chk2 phosphorylates approximately two residues per MCM2-7 complex of CMG under similar conditions (one each in MCM3 and MCM4 subunits) (35). The Chk1 proteins from *Drosophila* and humans phosphorylated MCM2-7 with similar efficiency in side-by-side titration experiments (Fig. S2, *B* and *C*). Showing the efficient overall phosphorylation of recombinant CMG by *Drosophila* Chk2 under these reaction conditions, the third target subunit of Chk2 in CMG, Psf2, undergoes an almost complete mobility shift as a result of phosphorylation at close to saturating Chk2 concentrations (Fig. S2*D*) (35).

These results show that the MCM2-7 replicative helicase motor complex is indeed a common phosphorylation target of Chk1 kinase in metazoans, suggesting that a conserved regulatory mechanism could be linked to this posttranslational modification pathway. We also found that Chk1 phosphorylated MCM subunits similarly in a free MCM2-7 as in the full CMG complex in both *Drosophila* (Fig. 2*C*) and mouse proteins (Fig. 2*D*). This suggests that the Chk1-dependent regulation potentially associated with this conserved phosphorylation site could, in principle, target the MCM2-7 core complexes both inside and outside of the CMG replicative helicase context.

**Figure 3:**
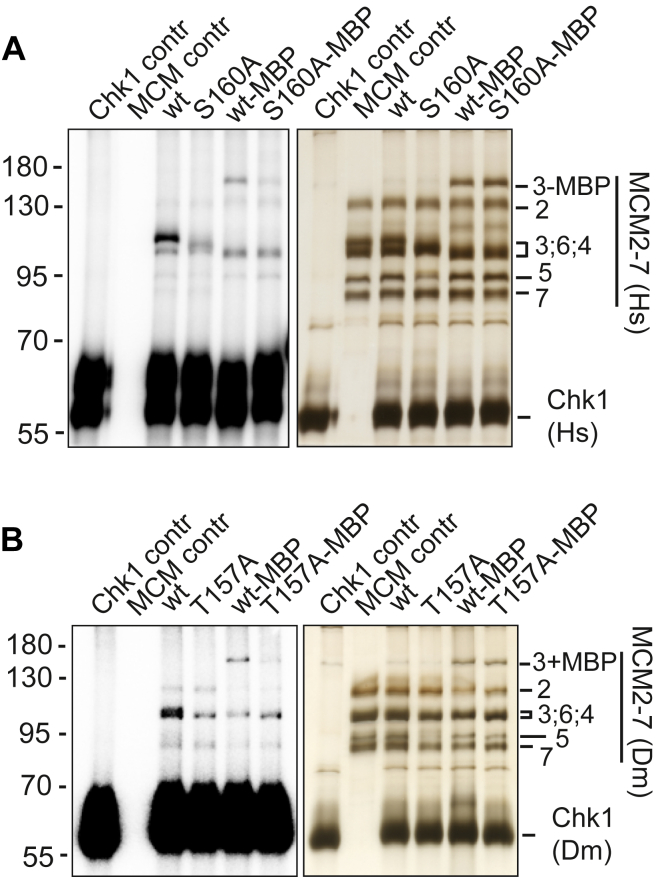
**Kinase assays with human (Hs) and Drosophila (Dm) MCM2-7 complexes carrying an S/T>A substitution at the characterized conserved position of the MCM3 subunit.** Silver-stained 10% SDS-PAGE protein gels are shown on the *right*, and PhosphoImager scan images of the same gels are shown on the *left*. “MBP” marks the lanes with MCM2-7 complexes carrying the N-terminal MBP tag on the MCM3 subunit. “contr” marks the control reactions with shown proteins alone. *A*, kinase assay with human wt and MCM3-S160A mutant complexes (75 nM MCM2-7; 1.9 μM Chk1). *B*, kinase assay with *Drosophila* wt and MCM3-T157A mutant MCM2-7 complexes (60 nM MCM2-7; 2 μM Chk1).

When concentrating on the specific subunits targeted by Chk1, most of the phosphorylation signal in our kinase assay radiographs overlapped with the region where the MCM subunits 3, 4, and 6 moved close to each other. This pattern was similar in human, mouse, and *Drosophila* proteins. Alanine substitution of the conserved Chk1 target residue S160 in humans and T157 in *Drosophila* MCM3 protein led to a significantly diminished Chk1-dependent phosphorylation signal in the comigrating MCM 3, 4, and 6 regions, although other MCM subunits were still clearly phosphorylated (Fig. 3, *A* and *B*). The same mutations also diminished the phosphorylation of the N-terminally MBP-tagged MCM3 subunits of human and *Drosophila* MCM2-7, which moved separately from the other MCM subunits (Fig. 3, *A* and *B*). The MBP tag also negatively impacted MCM3 phosphorylation, perhaps by physically interfering with the access of the kinase to its major target site in the N-terminal domain of the subunit. These data show that the described conserved residue of MCM3 is a major Chk1 kinase target site not only in humans but also in the *Drosophila* MCM2-7.

For additional detailed mapping of the Chk1 target residues in MCM2-7, we used mass spectrometry (MS) analysis of the *in vitro*–phosphorylated recombinant mouse and *Drosophila* MCM2-7 proteins. In these assays, we used ^18^O-labeled ATP as a phosphate donor, which helped to distinguish the phosphates added *de novo* by Chk1 (^18^O-labeled) from those that were already present in the recombinant proteins (nonlabeled) (Table S2). This approach confirmed the observations from radiography-based kinase assays, identifying the conserved S160 residue in mouse/T157 in *Drosophila* MCM3 as a primary Chk1 phosphorylation target site in the MCM2-7 complex. This analysis also identified several other, less efficient candidate Chk1 target sites in the MCM subunits. From these, the positions within the N-terminal nonstructured regulatory tail of MCM4 are the most significant hits.

Collectively, these data revealed that the MCM2-7 replicative helicase core complex is a common phosphorylation target for Chk1 kinase in metazoans and identified a conserved S/T residue in the MCM3 subunit as a major target residue for this kinase in the MCM2-7 complex.

### The candidate Chk2-dependent regulation pathway of the MCM2-7 replicative helicase core complex in *Drosophila* is unlikely to be conserved in metazoans or redundant with the Chk1-dependent pathway

**Figure 4:**
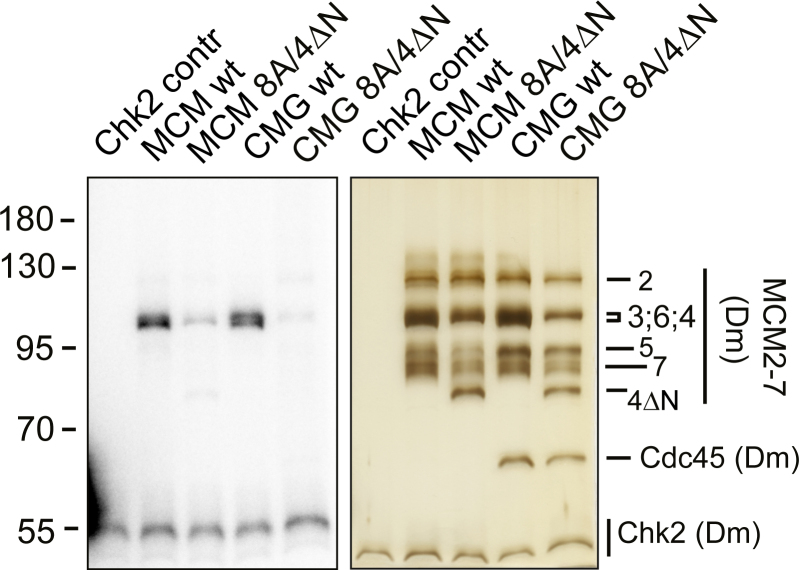
**Phosphorylation of MCM2-7 and CMG complexes by Drosophila Chk2 kinase**. Silver-stained 10% SDS-PAGE gel is on the *right*, and a PhosphoImager scan image of the same gel on the *left*, comparing the phosphorylation of the wt and MCM3-8A / MCM4-ΔN mutant MCM2-7 and CMG complexes (40 nM MCM2-7; 100 nM Chk2).

Chk2 kinase of *D. melanogaster* has been shown to inhibit the activity of CMG helicase *in vitro* by phosphorylating nonconserved unstructured regions in its three different subunits: C-terminal tail of Psf2, N-terminal tail of MCM4, and short stretch between the conserved AAA+ and C-terminal domains of MCM3 (35). In our kinase assays carried out with the recombinant proteins, deletion of the N-terminal tail of MCM4 in combination with point mutations of the eight identified target residues in MCM3 abolished almost all the phosphorylation of MCM subunits by *Drosophila* Chk2, both in the case of free MCM2-7 substrate and in the context of full CMG helicase (Fig. 4) (35). This indicated that, like in the case of Chk1, *Drosophila* Chk2 can target the MCM2-7 core complex both inside and outside the full replicative helicase context. As these Chk2 phosphorylation-deficient MCM2-7 and CMG mutant complexes still contained the conserved Chk1 target residue T157, this also indicated that Chk1 and Chk2 differentially target this position in *Drosophila*. These observations were supported by the mass spectrometry mapping of the Chk2 sites in *Drosophila* MCM subunits, which also failed to identify Thr157 as a target for Chk2 (35). The CMG targeting putative regulatory pathways dependent on Chk1 and Chk2 kinases are thus most likely nonredundant and could have different roles in the regulation of replicative helicase in *Drosophila*.

In contrast, the recombinant human and mouse Chk2 kinases displayed either very little or no MCM2-7 phosphorylation in side-by side kinase assays compared to the Chk1 kinases from the same organisms even at low μM kinase concentrations, while the phosphorylation of MCM2-7 by *Drosophila* Chk2 reached the saturation at around 300 nM kinase concentration in the same reaction conditions (Figs. S1*F* and S3, *A* and *B*). On the other hand, human and mouse Chk2 were able to efficiently phosphorylate Cdc25 and histone H1 control substrates, showing that the observed lower activity of these kinases towards MCM2-7 could not have been due to the inactive recombinant proteins (Fig. S1, *A–D*). Therefore, the MCM2-7 complex is not as effectively targeted by human and mouse Chk2 as in *Drosophila*. Similar observations were made in a previous report, in which the phosphorylation of MCM2-7 was tested side-by-side with human Chk1 and Chk2 kinases (42). In our assays with mixing and matching the kinases with the substrates, *Drosophila* Chk2 phosphorylated equally well its native as well as human (Fig. S3*C*) and mouse (Fig. S3*D*) MCM2-7 proteins, suggesting that the potential Chk2 kinase–docking determinants might also be present in the mammalian MCM2-7 subunits, but are lacking in the Chk2 kinase in these organisms. Thus, these docking determinants have either been lost in mammalian Chk2 proteins or acquired by *Drosophila* Chk2 during evolution.

The simplest model to incorporate these observations is that Chk2 kinase plays a specialized role in the regulation of MCM2-7 functions in *Drosophila*, which is not conserved in metazoans and is nonredundant with the Chk1-controlled MCM2-7 regulation pathway. It is consistent with the general paradigm, according to which ATR–Chk1 is the checkpoint kinase pathway involved in the control networks of genome replication, whereas the ATM-Chk2–controlled pathway performs distinct functions in the cell, primarily linked to the DNA double-stranded break response (43).

### The characterized conserved phosphorylation site is also found in the budding yeast MCM3, and mutations at this position lead to an altered growth phenotype in yeast

**Figure 5:**
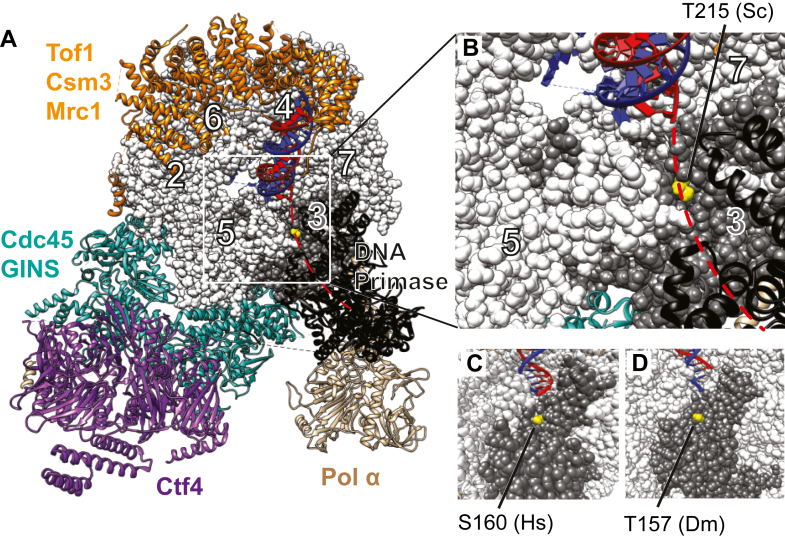
**Structure models showing the positioning of the characterized conserved phosphorylation site at the bottom of the ‘entry gate’ into the displaced DNA strand exit groove of CMG.** All panels present the *top* view of the N-terminal tier of the MCM2-7 ring of CMG. MCM2-7 is presented as a sphere model, and all other protein subunits of the complex as ribbon models. MCM3 subunits are colored *dark gray*, the conserved residue of interest is marked in *yellow*, and the leading and lagging DNA strands are colored *blue* and *red*, respectively. Images were created with UCSF Chimera software (73). *A*, structural model of the *Saccharomyces cerevisiae* partial replisome together with a DNA fork (PDB: 8B9B) (53). The individual components of the complex are colored as indicated on the panel. The dashed *red* line shows the expected path of the displaced strand from the DNA strand separation point towards the DNA Primase (ribbon model colored *black*) active center, which is supported by the cryoEM 3D classes from the same study. The displaced (lagging) strand exit channel region from this model (marked by a *white* box) is enlarged in panel (*B*). *C*, the lagging strand exit channel region from the structural model of the human partial replisome complex together with the DNA fork (PDB: 7PFO) (74). *D*, the lagging strand exit channel region from the structural model of *Drosophila* CMG together with an engaged DNA fork (PDB: 6RAX) (52).

Our homology alignment revealed that the identified metazoan Chk1 target residue in MCM3, as well as its surrounding amino acid sequence, is conserved in the budding yeast *Saccharomyces cerevisiae* MCM3 protein (Fig. 1). This sequence homology also translates well to the protein structure level, which will be discussed in greater detail in the following section (Fig. 5). The conserved position MCM3-T215 has been identified as a phosphosite in budding yeast, as demonstrated in a previous unbiased proteomic analysis of exponentially growing cells (44). Additionally, it has been demonstrated that MCM3-T215 is phosphorylated in prereplicative (pre-RC) complexes assembled *in vitro* in a Cdc7-Dbf4 (DDK) kinase–dependent manner, placing this phosphorylation event directly within the context of a crucial molecular step essential in genomic DNA replication (10).

It is unclear whether the budding yeast DDK itself is responsible for the phosphorylation of MCM3-T215 or if it is another protein kinase acting downstream of a DDK-dependent step during pre-RC formation. DDK has been well characterized as a key regulator of the MCM2-7 complex in the initiation of genomic DNA replication. However, even the recent detailed structural studies elucidating the phosphorylation mechanism of the MCM2-7 by DDK have not detected phosphorylation of the MCM3 T215 residue in yeast (10, 45, 46, 47) nor have any corresponding conserved residues in other organisms been identified as targets for DDK. Budding yeast Chk1, in contrast to its metazoan counterpart, is a nonessential kinase with no established links to S-phase regulation (48). Correspondingly, recombinant budding yeast Chk1 did not phosphorylate the MCM2-7 complex in our *in vitro* assays (Fig. S4*A*). From the other members of the Chk1/Chk2 family, Rad53—an ortholog of metazoan Chk2—plays a vital regulatory role in the genome replication of budding yeast, but it also appears to not phosphorylate MCM2-7 (Fig. S4). Additionally, Dun1, another checkpoint kinase in budding yeast that is paralogous to Chk2, is activated by Rad53 phosphorylation (49, 50, 51). We discovered that Dun1 phosphorylates at least two subunits of MCM2-7 in a Rad53-dependent manner; however, the phosphorylation signal did not comigrate with the MCM3 subunit band in the gel (Fig. S4). In conclusion, it is possible that another protein kinase is responsible for phosphorylating this conserved position in budding yeast MCM3 or that it is one of the discussed kinases but involving a more complex mechanism—perhaps dependent on cofactors such as DNA, other proteins, or pre-priming posttranslational modifications. Regardless of the specific kinase involved, the observed phosphorylation of this residue during the assembly of budding yeast pre-RC highlights its direct relevance to the genomic DNA replication process (10).

To examine the significance of the MCM3-T215 residue and its phosphorylation in budding yeast, we constructed mutant strains carrying either phosphomimetic aspartic acid (T215D) or inactivating alanine (T215A) substitutions in the genomic copies of *MCM3* and analyzed the phenotypic effects of these mutations on yeast growth. Both mutant strains formed colonies on agarose plates, with colony density and size indistinguishable from the wt strain. This suggested that the mutations do not have any drastic impact on growth. However, differences emerged in more sensitive co-growth assays (Fig. S5, *A* and *B*). In this setup, the wt strain consistently outgrew the *mcm3-T215A* mutant strain, indicating that the threonine residue at this position is required for optimal cell growth under normal, nonchallenging conditions. Conversely, the phosphomimetic *mcm3-T215D* strain outgrew the wt cells, suggesting that this substitution, which mimics the continuous phosphorylation at this position, gave a growth advantage to budding yeast cells during the span of the assay. These findings imply that the conserved MCM3-T215 residue and its phosphorylation may play a role in the molecular mechanisms influencing the growth of budding yeast cells.

We also investigated how T215A and T215D substitutions in MCM3 affected the growth of budding yeast under genotoxic stress conditions. In these experiments, both mutant strains produced colonies in response to treatment with varying doses of commonly used mutagens UV, hydroxyurea, and methyl methanesulfonate, exhibiting the same growth efficiency as the wt strain (Fig. S5*C*). Additionally, the MCM3-T215A and MCM3-T215D mutations did not rescue the sensitivity of Rad53, Mec1, or Chk1 checkpoint kinase knockout strains to hydroxyurea treatment (Fig. S5*D*). These findings suggest that the MCM3-T215 residue and its phosphorylation are not essential for the checkpoint response pathways activated by the genotoxic stress in budding yeast. However, it is still possible that here may be some nonessential redundant role played by the MCM3-T215 phosphorylation in these processes.

### The characterized conserved phosphorylation target residue is positioned at the bottom of the exit groove for the displaced DNA strand, and its phosphomimetic substitution affects the interaction of CMG with its forked DNA substrate

Numerous structural studies of CMG replicative helicases from humans, *Drosophila*, and budding yeast, in combination with *in vitro* biochemical analysis of this enzyme, have significantly advanced our understanding of how these ATPase-driven holoenzymes operate (6). Structural features critical for the molecular functions of CMG have turned out to be well conserved in eukaryotes from yeast to humans, with many core elements traceable back even to simpler archaeal homohexameric MCM helicase complexes. This information provided clues about the potential role of the characterized conserved phosphorylation site in the functioning of the CMG replicative helicase complex.

According to the current consensus, the double-stranded part of the unwinding substrate DNA can enter well into the N-terminal tier of the MCM ring of the CMG helicase before splitting into two single strands that serve as templates for the DNA polymerases. From these strands, the template for the leading DNA strand synthesis tracks into a central tunnel in the MCM2-7 ring, where it is likely pulled forward by the ATP hydrolysis–fueled coordinated action of several protruding beta-hairpin motifs of MCM subunits (52). The other, excluded single strand, which serves as a template for the lagging strand synthesis, seems to be most likely diverted into an exit channel lying between the zinc finger motifs of the MCM3 and MCM5 subunits on the surface of the N-terminal tier of the MCM2-7 ring. In the case of human and budding yeast CMG, this exit path has been supported by structural studies of CMG in a complex with Pol-alpha-primase (53), suggesting that it is indeed the path for the displaced strand that is productively engaged with the lagging strand synthesis machinery.

While examining the published structure models, we noticed that the characterized conserved phosphorylation target residue in MCM3 (S160 in humans and mice, T157 in *Drosophila*, and T215 in yeast) lies at the bottom of the entrance point into the displaced strand exit channel (Fig. 5). Therefore, we hypothesized that the addition of the negatively charged phosphate group by a protein kinase, mimicked by the aspartic acid substitution, could electrostatically repel the displaced DNA strand from its exit path in CMG. This, in turn, could weaken the interaction of CMG with the forked DNA substrate, thus affecting its helicase activity.

To test this hypothesis, we examined the *in vitro* helicase and DNA-binding activity of recombinant *Drosophila* wt, MCM3-T157A, or MCM3-T157D CMG complexes (Fig. S6*A*). Mixing the forked DNA template with purified CMG in the presence of ATP enables the entry of the 3′ single-stranded arm of the fork into the central channel of the MCM2-7 ring, likely by passing through the opening between the MCM2 and MCM5 subunits (“MCM2-5 gate”) (54). According to biochemical and structural studies, this yields what likely corresponds to a productive complex between the CMG helicase and its DNA substrate fork. When using nonhydrolyzable ATP analogs, like ATP-γS, the CMG is paused on the DNA fork, enabling the analysis of formed complexes. In the presence of hydrolyzable ATP, CMG starts to unwind the double strands by tracking the leading strand template in a 3′ to 5′ direction, enabling the analysis of the helicase activity of the complex (1, 2, 5, 55).

**Figure 6:**
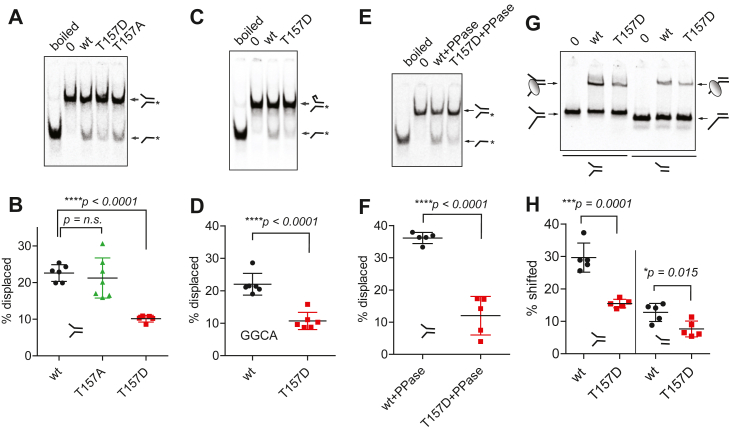
**Helicase assays and electromobility shift assays (EMSA) with recombinant Drosophila wt and mutant CMG protein complexes.***A*, helicase assay comparing the activities of wt, MCM3-T157A, and MCM3-T157D CMG on the forked DNA substrate with 50 bp double stranded region and 40 nucleotide long poly-T arms (2.5 nM). All the helicase and EMSA assays in this figure, with the exception of the experiments with phosphatase-treated CMG in panels (*E* and *F*), were carried out with 50 nM CMG concentration, which is close to the half saturation point (K_M_) of the helicase assay as shown in Fig. S6*C*. In all the helicase assay autoradiography images of this figure, the *arrows* mark the positions of double-stranded substrate and displaced oligo bands; “boiled” and “0” label the control lanes with heat-denatured substrate or without protein, respectively. The quantified data of the per cent of substrate processed from five replicates in the case of MCM3-T157D, seven in the case of MCM3-T157A, and six in the case of wt CMG are presented in panel (*B*). All the quantified data in this figure are presented as the replicates together with median value; error bars represent SD. *p* values were calculated using the unpaired two-tailed *t* test (n.s. – nonsignificant). *C*, helicase assay comparing the activities of wt and MCM3-T157D CMG on a forked DNA substrate (6 nM), where the 5′ displaced arm consists of “GGCA” repeats. The quantified data from six replicate experiments is shown in panel (*D*). *E*, helicase assay with bacteriophage λ phosphatase pretreated wt and MCM3–T157D CMG complexes (10 nM) and forked DNA substrate with poly-T arms (1 nM). The quantified data from five replicate experiments is shown in panel (*F*). *G*, EMSA comparing the binding of wt and MCM3-T157D CMG to a substrates containing either both poly-T single stranded DNA arms (first three lanes) or missing a displaced (5′) arm (last three lanes) (both substrates 5 nM). The quantified data from five replicate experiments is shown in panel (*H*).

We conducted *in vitro* helicase reactions using recombinant CMG and radioactively labeled DNA probes, employing polyacrylamide electrophoresis to separate the unwound radiolabeled strands from the unprocessed double-stranded probes. This analysis indicated that the MCM3-T157A mutation did not significantly affect the unwinding of the forked DNA substrate by CMG, showing that this conserved residue is not critical for the helicase activity of CMG. In contrast, the substrate unwinding was less efficient with MCM3-T157D mutant CMG, revealing that the phosphomimetic substitution at this position modulates the CMG helicase activity (Fig. 6, *A* and *B*). In all these helicase experiments involving small forked substrates, we pre-incubated CMG with DNA in the presence of ATP-γS before adding ATP to initiate the helicase activity (56, 57). This pre-incubation step enhances substrate unwinding by CMG (Fig. S6*B*), likely by facilitating the formation of a productive complex between the CMG helicase and its DNA substrate prior to the unwinding step. The phosphomimetic MCM3-T157D mutation also inhibited the unwinding of a similar DNA fork containing a 5′ flap made up of “GGCA” repeats, which forms a secondary structure that prevents the CMG complex from incorrectly loading onto the wrong single strand (Fig. 6, *C* and *D*) (56, 58). Furthermore, the MCM3-T157D mutation also impaired the helicase activity of CMG that had been pretreated with lambda-phosphatase to remove any phosphorylation from the surface of the recombinant complex (Fig. 6, *E* and *F*). This treatment has been shown to result in over a tenfold increase in the activity of the recombinant CMG helicase purified from a baculovirus expression system, suggesting the existence of additional phosphosites that can modulate CMG helicase activity (35). The inhibitory effect of the MCM3-T157D substitution on CMG helicase activity, as well as the lack of significant impact from the MCM3-T157A mutation, was confirmed using a DNA substrate formed by the radioactively labeled oligonucleotide with a 5′ single stranded flap annealed to the circular single-stranded genome of the M13 bacteriophage (Fig. S6, *D*–*G*). In these experiments, the ATP-γS pre-incubation step was omitted. Collectively, these results convincingly demonstrate that the phosphomimetic substitution at the conserved phosphorylation site in the MCM3 subunit inhibits CMG helicase activity.

In the discussed *in vitro* helicase assays, purified recombinant CMG must first productively bind to the DNA substrate before it can unwind it. Consequently, any mutations that impact the substrate DNA binding will also influence the results of the helicase assay. We assessed the binding of both wt and mutant CMG complexes to the radioactively labeled forked DNA substrate using the EMSA. In this assay, the CMG-bound radioactively labeled substrate molecules exhibit slower mobility during PAGE, allowing for the separation of the CMG-bound substrates from the unbound probe. In these experiments, the phosphomimetic MCM3-T157D mutant of the CMG complex demonstrated noticeably lower affinity for a DNA fork than the wt CMG (Fig. 6, *G* and *H*), while the MCM3-T157A mutant exhibited a similar affinity to that of the wt CMG (Fig. S6*H*). This indicates that the phosphomimetic substitution at this position interferes with the interaction between the CMG helicase and its forked DNA substrate, but the corresponding threonine residue itself is not critical for the interaction. We observed that the inhibitory effect of the MCM3-T157D mutation on the binding of the CMG complex was weaker, yet still statistically significant, when evaluated with a DNA substrate lacking a 5′ single-stranded arm (displaced stand) (Fig. 6, *G* and *H*). Thus, although the MCM3-T157D phosphomimetic substitution likely interferes with the interaction between the CMG helicase and the displaced strand of the DNA fork, it can still influence binding to a substrate that does not include this strand. This may be perhaps attributed to interference with the potential wrapping of the 3′ (leading) single strand into the displaced strand exit groove or to the repulsion caused by the negatively charged aspartate residue and the 5′ phosphate group at the junction of the double- and single-stranded regions of the substrate.

In conclusion, the findings from our *in vitro* helicase and DNA substrate–binding assays suggest that the phosphorylation of the characterized conserved serine/threonine residue within the MCM3 subunit, as simulated by the phosphomimetic substitution, can modulate the interaction between the CMG helicase complex and the unwinding substrate fork. Based on the structural information, this modulation is likely accomplished by interfering with the interactions involving the displaced strand of the DNA substrate fork.

## Discussion

In this study, we explored the checkpoint kinase–dependent molecular pathways that regulate eukaryotic genomic DNA replication by targeting the replicative CMG helicase. We discovered that the serine/threonine residue in the MCM3 subunit of CMG, previously identified as a target site for Chk1 protein kinase in human cells (36), is a conserved phosphorylation site in metazoans and potentially in some earlier diverged eukaryotes, including budding yeast. Drawing on the results from our *in vitro* DNA binding and helicase assays, along with available protein structure information, we propose that this phosphorylation site may modulate the interactions between the CMG helicase and an unwinding template fork. This modulation likely occurs because the phosphate group attached to this residue at the entry point of the displaced strand exit groove functions as an electrostatic “repellent” for the displaced DNA strand. The addition or removal of this phosphate group could enable precise adjustment of the interaction. Experiments with budding yeast showed that the mutations at this residue yield cell growth defects, which, together with the data from the previous analysis in human cells (36), are consistent with the conservation of the regulatory phosphorylation at this position, albeit the exact kinases involved and maybe also the exact downstream role of this pathway might differ in yeast compared to Metazoa. These observations suggest a novel conserved mechanism for phosphorylation-dependent regulation of the replicative helicase, the details and exact contribution of which to genomic DNA replication and maintenance will be elucidated in future studies.

The phosphorylated form of the characterized conserved residue is likely involved in the normal unperturbed process of genome replication. This phosphorylation site has been identified in multiple unbiased proteomic screens using unchallenged human, mouse, and rat cells (http://phosphosite.org), as well as in a proteomic study of exponentially growing budding yeast cells (44). Furthermore, in budding yeast, this specific position in MCM3 is one of the sites that undergoes phosphorylation in *in vitro*–assembled prereplicative complexes during the DDK-dependent phase (10), strongly indicating that its phosphomodification may be linked to normal replisome functions. A similar suggestion was made in the case of human cells, where the phosphorylated form of MCM3 at this position was found to localize mainly to the chromatin-enriched subcellular fraction in normally growing cells (36). The interaction between the CMG helicase and unwinding DNA fork must be optimal to ensure the best balance between helicase progression and its stability on the DNA template. Stronger interactions with either of the single strands of the unwinding fork can stabilize the helicase on its template but can also impede its progression. Weakening these interactions could assist in better sliding on the template, but an interaction that is too weak could also cause the helicase to slip on the template or even prematurely release the strands of unwinding DNA. Optimally coordinated interactions with both strands of the template fork are also critical for the functions of CMG helicase as a structural coordinator of the leading and lagging strand synthesis machineries of the replisome.

Supposing the phosphorylation of the conserved residue in question indeed corresponds to the optimal template interaction mode of the CMG helicase in normal replisome, the ability to switch between the phosphorylated and dephosphorylated state at this position has evolved in evolution in all likelihood because of some advantage that is provided by the capacity to carry out such a switch in response to certain conditions. The discussed phosphorylation target residue lies at the bottom of the displaced strand exit groove in CMG, where it is sterically protected by the displaced strand during DNA unwinding. Hence, if this site is phosphorylated in the normal replisome, its phosphorylation most likely takes place before the helicase is activated and replication starts. As a significant portion of the Chk1 is associated with chromatin in normal human cells (59, 60), its ability to phosphorylate MCM2-7 could be facilitated during the prereplication complex formation, considering the relatively low efficiency of the unassisted phosphorylation in our *in vitro* Chk1—MCM2-7 kinase assays compared to *Drosophila* Chk2. The masking effect of the displaced strand also means that phosphatases can remove the phosphate at this position only when the displaced strand is released, for example, after helicase stalling or when the CMG complex falls off the DNA entirely. Therefore, the dephosphorylation of this residue may be perhaps linked to molecular rescue mechanisms operating at stalled or collapsed replication forks. Consistent with this hypothesis, inducing replicative stress in human cells leads to reduced phosphorylation of chromatin-bound human MCM3 at the characterized conserved position. It coincides with the release of Chk1 from chromatin, which could keep MCM3 dephosphorylated at this position in conditions where replication stress leads to hyperactivation of the ATR–Chk1 kinase pathway to initiate the S-phase checkpoint response (36, 61). A possible mechanism by which the dephosphorylation of the characterized conserved residue could contribute to replication fork rescue could be by stabilizing the stalled CMG helicase on its forked DNA substrate until the paused replisome is ready to be restarted again.

Another possible regulatory mechanism for the described phosphorylation pathway involves facilitating the activation of the ATR-Chk1–dependent S-phase checkpoint response at stalled replication forks, as proposed previously (36). In normal eukaryote replisomes, DNA unwinding is tightly coupled with the synthesis of new strands. It might be achieved partly because of the significantly slower speeds of DNA unwinding by the CMG helicase alone than the DNA synthesis rates by the replisome, which prevents the helicase from escaping from the replisome (62, 63, 64). When the leading-strand polymerase stalls, for example, due to insufficient dNTP levels, CMG helicase can be decoupled from the replisome, generating RPA-covered single-stranded tracks of the unwinding DNA, a canonical activator of the ATR–Chk1 pathway. *Drosophila* MCM3-T157A mutant and wt CMG were able to unwind the DNA substrate forks more efficiently than the T157D phosphomimetic CMG in our *in vitro* helicase assays, suggesting that the decoupled CMG helicase that could be dephosphorylated at this position could facilitate the more efficient generation of ssDNA-RPA regions, in turn facilitating the activation of the S-phase checkpoint response. Consistent with this hypothesis, the average size of RPA foci induced in the nuclei of human cells treated with the DNA polymerase inhibitor aphidicolin was increased in MCM3-S160A mutant–expressing cells (36). This mutation has also been reported to lead to slightly longer replication tracks and faster BrdU incorporation in unchallenged human cells, suggesting faster replisome progression (36). The slower growth of the *mcm3-T215A* mutant yeast strain in our longer-term assays could be perhaps explained by the combined effect of replication stress caused by faster replication and sensitized S-phase checkpoint control, both caused by the same mutation. Based on the same hypothesis, the accelerated growth of *mcm3-T215D* yeast cells observed in our assays may be attributed to an optimal replication speed and a desensitized S-phase checkpoint response in these cells, which allows for uninterrupted progression through S phase despite the spontaneous fork stalling that occurs also during normal replication. Alternatively, the (Pre-RC) formation and/or activation could be more efficient in *mcm3-T215D* strain. This process coincides with the initial melting of the DNA template and could thus, in principle, be impacted by such posttranslational modifications that act on the interaction surface between the MCM2-7 and the forming DNA fork. It is also possible that phosphorylation at this conserved position has distinct regulatory roles in yeast compared to Metazoa.

Our data demonstrate that phosphorylation-dependent regulatory pathways can directly target the interactions between CMG replicative helicase and its DNA substrate. By implicating the ATR–Chk1 kinase pathway in this regulation, these observations further consolidate the role of this pathway as one of the central hubs in the regulation of genomic DNA replication in metazoans. In addition to Chk1, additional kinases may be involved in regulation pathways targeting the characterized conserved phosphorylation site in MCM3. For example, the human death–associated kinase DAPK can phosphorylate the MCM3-S160 position, although the exact role of this kinase in the regulation of CMG helicase remains undetermined (65). In budding yeast, it may not be Chk1 but an entirely different kinase phosphorylating the conserved MCM3-T215 position, reflecting the mechanistic differences in the replication control by checkpoint kinases in yeast compared to Metazoa.

Finally, it is interesting to note that the budding yeast Dia2 and metazoan LRR1 specificity factors of the ubiquitin ligase complex that triggers the ubiquitination-dependent disassembly of CMG during replication termination bind to the region in MCM2-7 overlapping with the displaced strand exit channel (66). Overall, the conserved phosphorylation pathway characterized here opens up new perspectives for future studies to establish the details and exact role of this regulatory mechanism in molecular networks that ensure the correct and timely duplication of genomic DNA.

## Experimental procedures

### Phylogenetic sequence alignment of MCM3 proteins

MCM3 protein sequences were retrieved from the Kyoto Encyclopedia of Genes and Genomes database (http://www.kegg.jp). Multiple sequence homology alignment was performed using the Clustal W option integrated into the Kyoto Encyclopedia of Genes and Genomes query results page. The alignment output was edited with Jalview software (67), using the Blosum color scheme option to express the relative conservation at each position.

### Purification of recombinant proteins from the baculovirus expression system

Most of the recombinant proteins used in this study were purified using a baculovirus expression system. Baculoviruses expressing mouse wt MCM2-7 subunits (68) and following *Drosophila* proteins have been previously characterized: wt CMG subunits (1), flag-MCM3-8A (carries the Ala substitutions in positions S720, S725, S734, S735, S739, T742, S743, and T744), MCM4-ΔN, Chk1, and Chk2 (35). New baculoviruses were constructed for expressing the following proteins: mutant MCM3 proteins with substitutions in the characterized conserved phosphorylation site (*Drosophila* MCM3-T157A and T157D, mouse and human MCM3-T160A); human MCM2-7 subunits, Chk1, and Chk2; mouse GINS subunits (GINS3 with C-terminal Strep affinity tag), Cdc45, Chk1, and Chk2; and budding yeast Rad53. The same D>N substitution of a conserved catalytic residue in the kinase subdomain VIb was introduced in all kinase-defective constructs. All the protein kinases and MCM3 proteins were expressed with the N-terminal FLAG affinity tag, and the human and *Drosophila* FLAG-MBP-MCM3 proteins additionally carried the maltose-binding protein tag between the FLAG and MCM3 coding sequences. For constructing the individual baculoviruses, the cDNAs were cloned into a pFastBac1 donor vector, from which the expression cassette was recombined into the baculovirus genome using the Bac-to-Bac protocol and reagents (Invitrogen, Thermo Fisher Scientific). All point mutations were introduced using standard PCR-based site-directed mutagenesis methods. The ORFs of all final vector constructs were verified by complete sequencing.

All protein kinases were purified following a previously characterized FLAG affinity protocol (1, 35). *Drosophila*, mouse, and human MCM2-7, as well as the *Drosophila* and mouse CMG were purified following a previously characterized protocol combining FLAG affinity and Mono Q ion exchange (Cytiva) chromatography steps (FLAG tag in MCM3) (1). In the case of the mouse CMG complex, an additional Strep-Tactin (IBA Lifesciences) affinity step preceded the FLAG affinity step (Strep-tag in GINS3). The same protocol was used for Strep-tag as for FLAG-tag–dependent affinity purification, except that the elution buffer contained 5 to 10 mM desthiobiotin instead of the FLAG peptide. The lambda phosphatase pretreatment of *Drosophila* CMG complexes, together with the following re-purification of the CMG, was carried out as described previously (35).

The final concentrations of all purified recombinant proteins were determined by the densitometry analysis of PageBlue or SYPRO Orange (Thermo Fisher Scientific)–stained PAGE gels, where the protein samples were run together with the bovine serum albumin protein concentration standards. PageBlue-stained gels were scanned using Epson Perfection V800 photo scanner and the densitometry analysis was carried out using ImageJ software (69); the SYPRO Orange–stained gels were scanned using Typhoon fluoroimager (GE Healthcare) and ImageQuant TL software was used for the densitometry analysis.

### Phosphorylation assays

All purified recombinant proteins were dialyzed into reaction buffer A (25 mM Hepes–NaOH pH 7.6, 100 mM sodium acetate, 10 mM magnesium acetate, 10% glycerol) supplemented with 1 mM DTT before preparing aliquots and snap freezing in liquid nitrogen for long-term storage at −80 °C. The *in vitro* kinase reactions with recombinant kinases and MCM2-7 or CMG were carried out in the same buffer, supplemented with 300 μM ATP spiked with γ-^32^P ATP (approximately 2.5 μCi per reaction) (Hartmann Analytic GmbH, Cat. # SRP-501) and 250 μg/ml insulin (Merck KGaA, Cat. #I9278) as a crowding agent. Bovine histone H1, used in some kinase tests, was purchased from Sigma-Aldrich (Cat. # 14-155). The kinase reactions were incubated for 20 min at 30 °C and stopped by adding 6X SDS-PAGE sample buffer (60% glycerol, 300 mM Tris–Cl (pH 6.8), 12 mM EDTA, 12% SDS, 0.05% bromophenol blue, 864 mM β-mercaptoethanol). The proteins were resolved using 10% polyacrylamide gel (29:1 acrylamide:Bis) electrophoresis in a standard Tris-Glycine running buffer and stained with PageBlue Protein Staining Solution (Thermo Fisher Scientific) or silver staining. For silver staining, the gels were sequentially incubated in 50% methanol, 5% methanol, 32 μM DTT, and 1 mg/ml silver nitrate solutions, 10 min each step, with quick rinses with distilled water after the DTT and silver nitrate incubation. Protein bands in the gel were then developed with 30 mg/ml sodium carbonate and 6.7 mM formaldehyde solution, and the reactions were stopped with 5 mg/ml citric acid solution. The stained gels were air-dried between two sheets of cellophane and exposed to the autoradiography (X-ray) film (CP-BU NEW from Agfa HealthCare NV) or scanned with a Typhoon PhosphorImager scanner (GE Healthcare) to reveal the phosphorylated protein bands. ImageQuant TL software was used to quantify the radioactive phosphorylated protein bands. All the conclusions about the relative phosphorylation of target substrates by different kinases or the effect of different mutations in the kinase or substrate proteins on the phosphorylation are based on the data from three or more independent kinase experiments.

For the phosphorylation assays with Chktide peptide substrate (Sigma-Aldrich) derived from the Chk1/Chk2 target site in Cdc25 protein, the reactions were carried out with 700 nM Chk1 or Chk2, spotted onto pieces of phosphocellulose paper, washed with 75 mM orthophosphoric acid, and air dried. The radioactive phosphorylation signals were then scanned using the GE Healthcare Typhoon PhosphorImager and quantified using ImageQuant TL software. GraphPad Prism software package (Dotmatics) was used to plot the Michaelis–Menten curves and calculate the kinetic parameters. Preliminary time-course experiments were carried out to ensure that the reaction conditions were optimal for steady-state analysis. Here, two replicate experiments were carried out, confirming the previous observations about the feasibility of the recombinant approach for producing the active kinase proteins.

### Proteomics sample preparation and LC-MS/MS

The LC-MS/MS analysis was carried out in the Proteomics Core Facility of the Institute of Technology, University of Tartu, Estonia. For the MS mapping of the Chk1 phosphorylation sites in mouse MCM2-7 and *Drosophila* CMG, the kinase reactions were carried out as described above, except that 1 mM [^18^O]ATP (Cambridge Isotope Laboratories) was used and insulin was omitted from the reactions. LysC and trypsin digestion of phosphorylated proteins and subsequent sample preparation for LC-MS/MS analysis were performed as described previously (70).

The ^18^O-labeled phosphosites were mapped in two experiments. In the first analysis, the digested peptides were separated on a 15 cm × 75 μm ID emitter column (New Objective) packed with 3 μm ReproSil-Pur C18AQ beads (Dr Maisch), attached to an Agilent 1200 series nano-LC. A linear 120 min 8 to 45% gradient of solvent B at a flow rate of 250 nl/min was used to elute the peptides from the column (column buffer 1: 0.1% formic acid; column buffer 2: 80% acetonitrile + 0.1% formic acid). The peptides were detected using an LTQ Orbitrap XL mass spectrometer (Thermo Fisher Scientific). Each 300 to 1800 m/z MS scan at a resolution setting 60 0000 was followed by MS/MS analysis of up to five most intense peaks. In the second analysis, the peptides were separated using an Ultimate 3000 RSLCnano system (Dionex) equipped with a C18 trap column (Dionex) and the same analytical C18 emitter column as in the first run, using a linear 90 min 8 to 40% gradient of solvent B at a flow rate of 200 nl/min. The peptides were detected using a Q Exactive Plus mass spectrometer (Thermo Fisher Scientific). Each 350 to 1400 m/z MS scan at a resolution setting of 70,000 was followed by MS/MS analysis of up to 10 most intense ions. Only charge states over +1 were analyzed in both experiments.

Raw MS data files were processed using the MaxQuant software package (version 2.2.0.0) (71). Phospho (STY) and manually added phospho 18O (STY) modifications were set as variable modifications, and the default search parameter settings were used. Peptide identification searches were performed against the compiled subset of the appropriate kinase and substrate protein reference sequences downloaded in September 2023 from the UniProtKB database (https://www.uniprot.org/).

### EMSA and helicase assays

The bacteriophage M13 genomic DNA–based substrates used in the helicase assays in Fig. S6, *D*–*G* were prepared as described previously (2). Briefly, the T4 polynucleotide kinase (New England Biolabs) and γ-^32^P ATP (Hartmann Analytic GmbH, Cat. # SRP-501) were used to radioactively label the 5′ end of a 5′30T40mer oligonucleotide (T)_30_GGTTTTCCCAGTCACGACGTTGTAAAACGACGGCCAGTG, which was then annealed to a single stranded circular M13 genomic DNA. Illustra microspin G-25 and S-400 spin columns (Cytiva) were used for separating the labeled oligonucleotides from the excess γ-^32^P ATP or double-stranded substrates from the non-annealed oligonucleotide, respectively. The final M13-based substrate has a 39 bp double stranded region and 30 nucleotide single stranded 5′ poly-T flap.

The oligonucleotide-based short forked substrates used in EMSA and helicase assays in Figs. 6 and S6, *B* and *C* were prepared essentially as described in (57), except that we used radioactive labeling instead of the fluorescent label. These substrates carried the same radioactively labeled strand with a 3′ 40 nucleotide poly-T flap:

GAGACCGAACGATCCTGTAATGTCCTAGCAAGCCAGAATTCGGCAGCGTC(T)_40_

This was annealed to the following 50-nucleotide complementary oligonucleotide to result in a substrate lacking the 5′ flap:

GACGCTGCCGAATTCTGGCTTGCTAGGACATTACAGGATCGTTCGGTCTC

In the case of the forked substrates with 5′ poly T or GGCA flaps, the complementary oligonucleotide additionally carried the (T)_40_ or (GGCA)_10_ sequences, respectively, at its 5′ end. Thirty picomoles of the 3′ poly-T oligonucleotide were end-labeled with the T4 polynucleotide kinase (New England Biolabs) using γ-^33^P ATP (Hartmann Analytic GmbH, Cat. # SRF-301) as a phosphate donor. Illustra microspin G-25 spin column (Cytiva) was used to separate labeled oligonucleotide from the excess γ-^33^P ATP, and 10 pmol of the labeled oligonucleotide was mixed with 100 pmol of the desired complementary strand in an annealing buffer containing 20 mM Hepes pH 7.6, 50 mM NaCl, and 3 mM MgCl2. The annealing reactions were heated to 98 °C for 3 min and then left to cool down to a room temperature slowly. Double-stranded forked DNA substrates were separated from the nonannealed oligonucleotides using 4% acrylamide gel electrophoresis (29:1 acrylamide to bis-acrylamide ratio) in 1× TBE buffer; the double-stranded substrate bands were cut out and eluted from the gel, precipitated with ethanol, and dissolved in reaction buffer A (25 mM Hepes–NaOH pH 7.6, 100 mM sodium acetate, 10 mM magnesium acetate, 10% glycerol).

In the CMG-binding reactions (EMSA), the prepared oligonucleotide-based forked substrates (5 nM) were incubated with purified CMG complex for 30 min at 30 °C in buffer A supplemented with 100 μM ATP- γS, 1 mM DTT, and 250 μg/ml insulin as a crowding agent. The protein–DNA complexes were then loaded onto a 4% polyacrylamide gel (60:1 acrylamide to bis-acrylamide ratio) supplemented with 5% glycerol and 6 mM magnesium acetate and separated by electrophoresis in 0.5× TBE with 6 mM magnesium acetate buffer at 4 °C. The gels were fixed in 20% methanol and 10% acetic acid for 10 min and dried to a piece of Whatman paper in a vacuum drier at 80 °C.

In the EMSA assays quantified in Fig. S6*H*, the same methods were used, except that the reactions were incubated for 20 min before loading on the gel and a different substrate fork was used, which was annealed from the following oligonucleotides:

− 5′-CACTGGCCGTCGTTTTACAACGTCGTGACTGGGAAAACC(30T)-3′
− 5′-(30T)GGTTTTCCCAGTCACGACGTTGTAAAACGACGGCCAGTG-3′

The helicase reactions with the M13-based substrates were performed in a reaction buffer A supplemented with 300 μM ATP, 1 mM DTT, and 250 μg/ml insulin as a crowding agent. The reactions were incubated for 30 min at 30 °C. In the helicase reactions with the short forked DNA substrates (Figs. 6, *A*–*F* and S6, *B* and *C*), the radiolabeled substrates were pre-incubated with purified CMG complexes for 20 min at 30 °C in the buffer A supplemented with 10 μM ATP- γS, 1 mM DTT, and 250 μg/ml insulin. One millimolar of ATP was then added to start the unwinding, and the reactions were incubated for additional 20 min at 30 °C. All the helicase reactions were stopped by adding SDS (0.1%) and EDTA (20 mM). The reaction products were separated by 8% PAGE (29:1 acrylamide: Bis ratio) in 1× TBE running buffer + 0.1% SDS and the gels were dried to a piece of Whatman paper in a vacuum drier at 80 °C.

The dried EMSA and helicase assay gels were scanned using a GE Healthcare Typhoon PhosphorImager. ImageQuant TL software was used to quantify the scanned images, and statistical analysis of the quantified data was carried out with the GraphPad Prism software (Dotmatics). The unpaired two-tailed *t* test was used to calculate the *p*-values and validate the statistical significance when comparing the datasets. The DNA-binding activity of CMG in EMSA assays was expressed as a % of substrate shifted into discreet slower moving bands, and the helicase activity as a % of substrate unwound according to the faster moving displaced radioactive oligonucleotide band.

## Data availability

All the data underlying this article is either presented within the manuscript and Supporting information or will be shared on reasonable request to the corresponding author. The mass spectrometry proteomics data of the Chk1 phosphosite mapping in MCM subunits have been deposited to the PRIDE database under accession numbers PXD049048 and PXD049049.

## Supporting information

This article contains Supporting information (75).

## Conflicts of interest

The authors declare that they have no conflicts of interest with the contents of this article.
