# Supplementary material for: A conserved phosphorylation mechanism for regulating the interaction between the CMG replicative helicase and its forked DNA substrate
Source: J Biol Chem. 2025 Mar 14;301(4):108408. doi: 10.1016/j.jbc.2025.108408 (PMC12018195; doi:10.1016/j.jbc.2025.108408)
Supplement: Supporting information [file mmc1.pdf]

Supporting information for:

A conserved phosphorylation mechanism for regulating the interaction between the CMG replicative helicase and its forked DNA substrate

Sandra Koit [1*], Nele Tamberg [1,3*], Allan Reinapae [2], Lauri Peil [1,3], Arnold Kristjuhan [2], and Ivar Ilves [1,4]

[1] Institute of Technology, University of Tartu; Nooruse 1, Tartu 50411, Estonia

[2] Institute of Molecular and Cell Biology, University of Tartu; Riia 23, Tartu 51010, Estonia

[3] Present address: Icosagen Cell Factory OÜ; Eerika tee 1, Õssu, Kambja vald, Tartu maakond, 61713, Estonia

[4] Corresponding author, ivar.ilves@ut.ee

[*] These authors contributed equally to this work

Includes:

Experimental procedures related to the Figure S5

Table S1

Legend to the Table S2

Figures S1-S6

Experimental procedures related to the Figure S5

Recombinant protein purification from yeast

Genes encoding N-terminal FLAG-tagged budding yeast Chk1 proteins were cloned under the control of a galactose-inducible GAL1-10 promoter and integrated into the ADE2 locus in the strains AKY2401 (WT Chk1) and AKY2424 (kinase-defective Chk1 D142N mutant). Yeast cultures (400 ml, approximately 1 × 10^7 cells per ml) were induced for 7 h in galactose-containing growth medium, the last two hours in the presence of 0.05% MMS to activate the checkpoint response. The cells were harvested, washed with water, and snap-frozen in liquid nitrogen. The frozen yeast pellets were crushed together with 5 ml of frozen 'popcorn' of buffer C (25 mM Hepes - KOH pH 7.6, 10% glycerol, 1 mM $MgCl_2$, 2 mM β-mercaptoethanol, 20 mM beta-glycerophosphate, 10 mM NaF) with 50 mM KCl and protease inhibitor cocktail (Roche). Three 3-min bursts with a frequency setting of 30 s−1 were applied in an MM400 Retsch ball mill, and the grinding jars were cooled in liquid nitrogen after each burst. The extract was thawed (all the following steps on ice or at 4 °C), and an additional 5 ml of buffer C with 50 mM KCl and protease inhibitors was added together with benzonase at 50U/ml final concentration (MilliporeSigma Novagen). After incubation for 1h, the KCl concentration was adjusted to 250 mM, and the extract was mixed for 20 min before clarifying by centrifugation at 50,000 x g for 30 min. 100 µl of Anti-FLAG M2 agarose beads (Sigma Aldrich) were added to the clarified extract and incubated for 3 h with constant mixing. Beads were collected by centrifugation, transferred to a disposable chromatography column, washed four times with buffer C + 500 mM KCl (all washing steps with at least ten column volumes of wash buffer), and once with buffer C + 250 mM KCl, both buffers supplemented with 0.4 mM PMSF. The column was then washed once with buffer C + 100 mM KCl (without β-glycerophosphate and NaF), and the proteins were eluted with the same buffer containing 200 µg/ml FLAG peptide. Five 100 µl fractions were collected into separate tubes and snap-frozen in liquid nitrogen for a long-term storage at -80°C.

The budding yeast MCM2-7 + Cdt1 complex was purified as described previously (Frigola, J., Remus, D., Mehanna, A., and Diffley, J. F. (2013) ATPase-dependent quality control of DNA replication origin licensing. Nature. 495, 339–343), with the following modifications. The cells were ground in a Retch ball mill, as in the case of the Chk1 protocol above, and treated with 75 U/ml benzonase before the affinity purification step. The K glutamate concentration was kept at 100 mM throughout the protocol. yJF38 strain expressing the MCM2-7+Cdt1 proteins was kindly provided by John Diffley (The Francis Crick Institute, London, UK).

Recombinant FLAG-tagged budding yeast Dun1 kinase was kindly provided by Marko Lõoke and Mart Loog (Institute of Technology, University of Tartu, Estonia).

Yeast competitive growth assay.

Yeast strains expressing the Mcm3 mutants T215A (AKY1993) or T215D (AKY1995) were constructed by replacing the native *MCM3* coding sequence with *mcm3* point mutations in codon T215. In addition, to distinguish between strains in the mixed cultures, the *BAR1* gene was replaced with the *LEU2* marker gene in *mcm3-T215* mutant strains and with the *hphMX6* gene in the wild-type MCM3 strain (AKY1095). The strains were grown in 25 ml YPD medium overnight at 30°C, diluted into fresh media, and grown further for five hours to the mid-log phase (approximately 5x10^7 cells/ml), and the cells were counted. 20,000 cells of the wild-type *MCM3* strain were mixed with equal amounts of either the *mcm3-T215A* or *mcm3-T215D* strain in 20 ml of YPD media (final concentration 2000 cells/ml). The co-cultures were

grown in Erlenmeyer flasks in a shaker for 24 h at 30°C, and new dilutions were made in fresh media. The same procedure was repeated for two weeks. After every dilution, 150 µl of the culture was seeded on YPD, SD-leu, and YPD-hygromycin-B agar plates to determine the proportion of wt/mutant Mcm3 strains in the co-culture. The plates were incubated for two days at 30°C, photographed, and the colonies were counted using ImageJ software.

Table S1. Yeast strains used in this study.

**Strain**	**Genotype**	**Source**
AKY1095	*W303; MAT A mcm3::MCM3-natMX6 bar1::hphMX6*	This study
AKY1993	*W303; MAT A mcm3::mcm3-T215A-natMX6 bar1::LEU2*	This study
AKY1995	*W303; MAT A mcm3::mcm3-T215D-natMX6 bar1::LEU2*	This study
AKY2437	*W303; MAT A sml1::klURA3*	This study
AKY2439	*W303; MAT A sml1::klURA3 mcm3::mcm3-T215A-natMX6*	This study
AKY2441	*W303; MAT A sml1::klURA3 mcm3::mcm3-T215D-natMX6*	This study
AKY2451	*W303; MAT A sml1::klURA3 rad53::spHIS5*	This study
AKY2443	*W303; MAT A sml1::klURA3 rad53::spHIS5 mcm3::mcm3-T215A-natMX6*	This study
AKY2445	*W303; MAT A sml1::klURA3 rad53::spHIS5 mcm3::mcm3-T215D-natMX6*	This study
AKY2370	*W303; MAT A sml1::klURA3 mec1::LEU2*	This study
AKY2378	*W303; MAT A sml1::klURA3 mec1::LEU2 mcm3::mcm3-T215A-natMX6*	This study
AKY2380	*W303; MAT A sml1::klURA3 mec1::LEU2 mcm3::mcm3-T215D-natMX6*	This study
AKY2405	*W303; MAT A chk1::TRP1*	This study
AKY2415	*W303; MAT A chk1::TRP1 mcm3::mcm3-T215A-natMX6*	This study
AKY2417	*W303; MAT A chk1::TRP1 mcm3::mcm3-T215D-natMX6*	This study
AKY2401	*W303; MAT A ade2::GAL-Flag-chk1::ADE2 pep4::kanMX6*	This study
AKY2424	*W303; MAT A ade2::GAL-Flag-chk1-D142N::ADE2 pep4::kanMX6*	This study

Legend to the Table S2. Microsoft Excel table presenting the Chk1 phosphorylation sites identified in mouse (Mm) MCM2-7 and Drosophila (Dm) CMG using LC-MS/MS analysis. O18-labeled ATP was used as a phosphate donor to distinguish *de novo* phosphorylated sites from those already modified in these baculovirus-expressed recombinant proteins. The Mm and Dm data are presented as separate sheets. In both cases, two different LC-MS/MS analysis runs were performed as described in the Materials and Methods section (sample names 'Mm1' and 'Mm2' in case of Mm analysis, and 'Dm1' and 'Dm2' in case of Dm analysis). The identified phosphosites are grouped according to the protein subunit of origin and are presented in the order they are found within the protein. The rows containing the phosphorylation data of the Mm MCM3-S160 and Dm MCM3-T157 sites are presented in red.

The following most relevant identification parameters extracted from the Phospho 18O (STY)Sites output table of the MaxQuant analysis are presented:

- Intensity – the sum of extracted ion chromatogram data of all isotopic clusters associated with the identified peptide, which correlates with its quantity in a sample. Intensity <sample name> column shows the summed value from all the differentially modified peptides associated with the site. Intensity <sample name>_1, _2, and _3 columns present quantitative information for the identified phosphosite coming from either a mono-, di or tri-phosphorylated peptides, respectively. Intensity value "0" or missing value means that the signal fell below the quantitation threshold set by the default settings in MaxQuant.

- Ratio mod/base <sample name> – the ratio of modified site to unmodified site in the sample based on intensity measurements.

- Phospho 18O (STY) Probabilities – the best localization probabilities of the identified phosphorylation sites shown within a peptide sequence (from 0 to 1, where 1 is the best match). Localization prob <sample name> columns show the localization probability in a particular sample.

- Score <sample name> – highest MaxQuant Andromeda peptide spectrum match identification score associated with the indicated modification site in a particular sample.

- PEP <sample name> – lowest posterior error probability score of identification (probability of wrong identification) for a peptide spectrum matches associated with the indicated phosphosite in a particular sample.

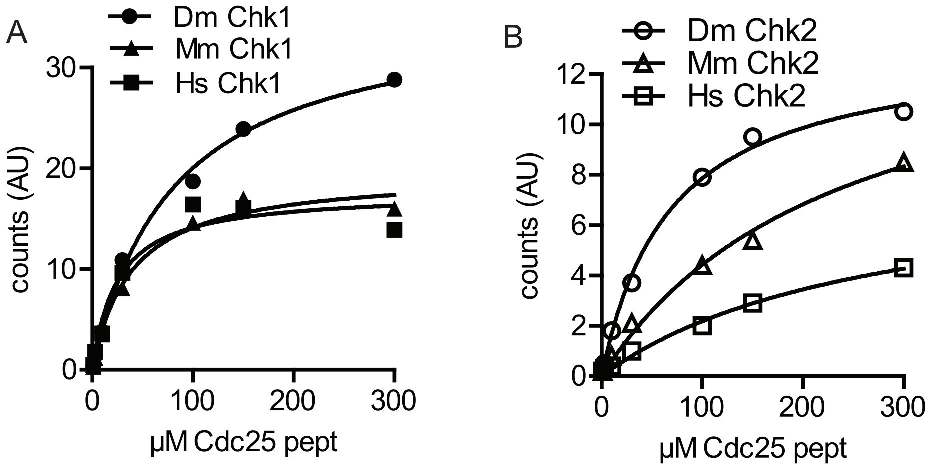

C

	Dm Chk1	Mm Chk1	Hs Chk1	Dm Chk2	Mm Chk2	Hs Chk2
**Vmax**	36 ±2	20 ±1	18 ±2	13 ±1	15 ±2	8 ±1
**Km (µM)**	81 ±10	39 ±9	25 ±10	69 ±8	238 ±52	275 ±76

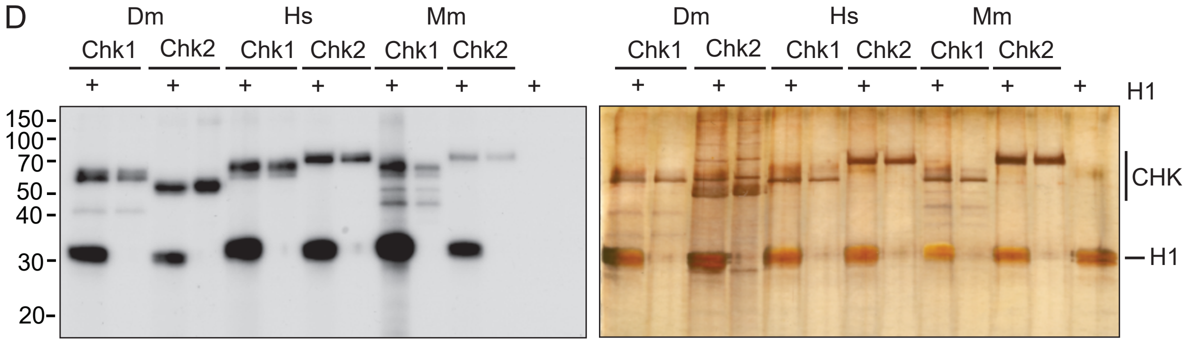

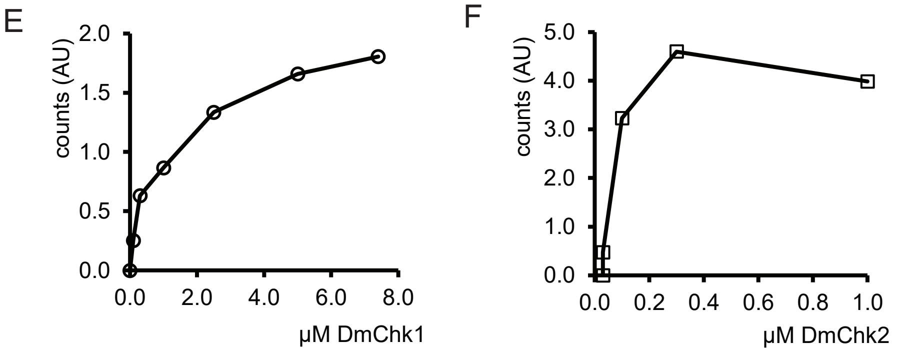

Figure S1. Testing of the activities of baculovirus-expressed purified recombinant Chk1 and Chk2. (A-B) Kinase assays measuring the rate of phosphorylation of a Cdc25C peptide substrate by the Drosophila (Dm), mouse (Mm), and human (Hs) recombinant Chk1 (A) and Chk2 (B) kinases. Y axis shows the relative count of radioactive phosphate transferred in arbitrary units. All the reactions were performed in parallel using the same radioactive ATP-spiked ATP mix, which allowed for the comparison of the saturation curves. Enzymatic parameters calculated from these data as Michaelis-Menten best-fit values are shown in (C), together with standard errors. (D) In vitro kinase assays comparing the phosphorylation of histone H1 (650 nM) by recombinant Chk1 and Chk2 kinases (100 nM each). The silver-stained 10% PAGE-SDS gel is shown on the right and the autoradiography film image of the same gel is shown on the left. (E-F) Quantified phosphorylation signals from the MCM3 region of the PhosphorImager scan image from the Fig. 2B.

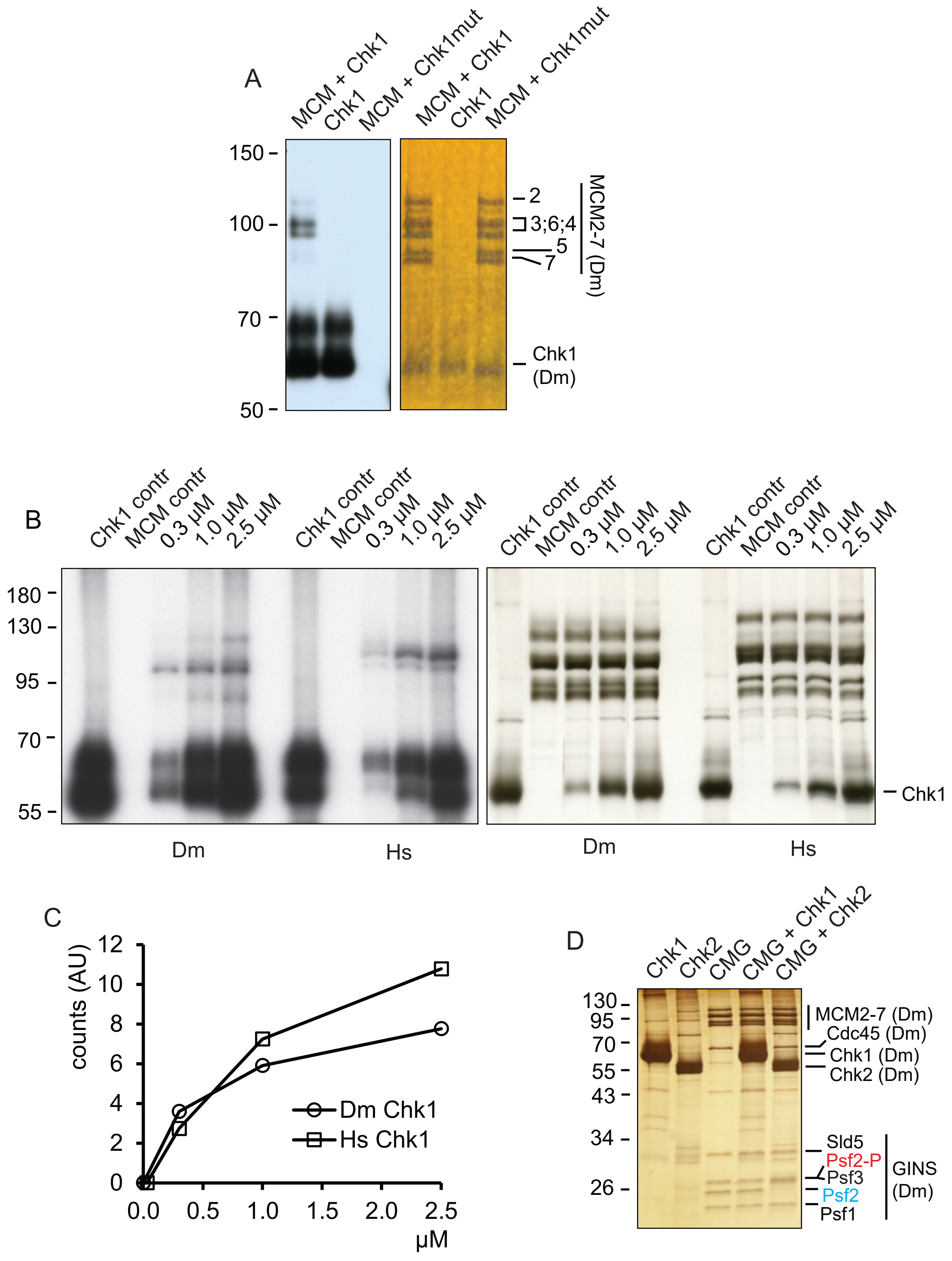

Figure S2. Kinase assays with Drosophila (Dm) and human (Hs) proteins. (A) Phosphorylation of MCM2-7 (100 nM) with wt and kinase-defective Chk1 (‘Chk1mut’). Here and in (B), the silver-stained 10% SDS-PAGE protein gels are shown on the right, and autoradiography film images of the same gels are shown on the left. (B) Comparison of MCM2-7 (100 nM) phosphorylation by indicated concentrations of Drosophila and human Chk1. Quantified phosphorylation signals from the MCM3 region of the PhosphorImager scan are presented on (C). (D) Chk2 phosphorylation-dependent mobility shift of the Psf2 subunit of Drosophila CMG at the used near saturating kinase concentration (2.2 µM Chk1; 500 nM Chk2; 30 nM CMG). The non-phosphorylated and phosphorylated Psf2 bands in the presented silver-stained 12% PAGE-SDS protein gel are marked in blue and red, respectively.

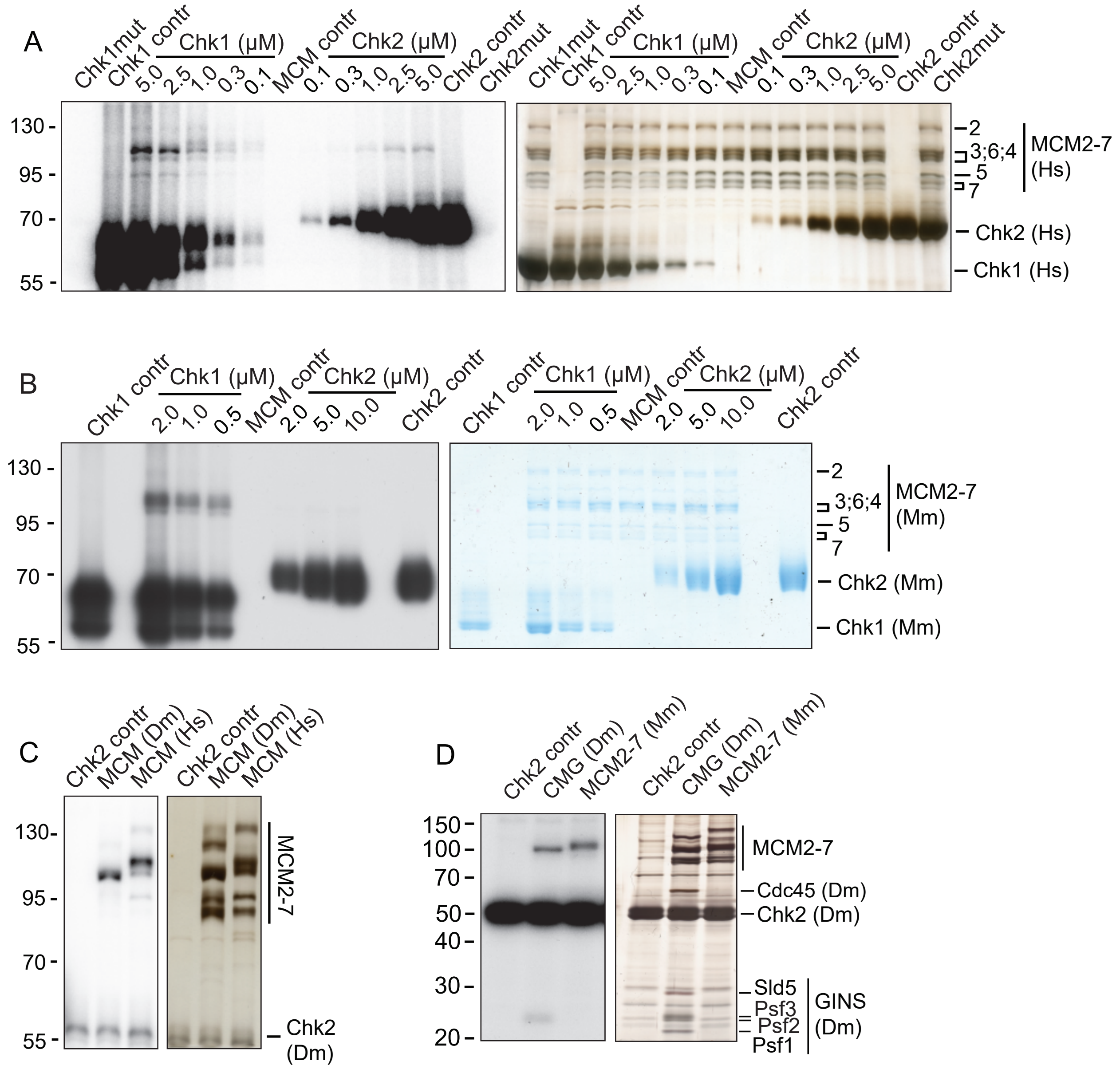

Figure S3. Kinase assays using Drosophila (Dm), human (Hs), and mouse (Mm) proteins. 10% SDS-PAGE protein gels stained with either Coomassie brilliant blue (B) or silver staining protocol (all other panels) are shown on the right, and autoradiography film images of the same gels are shown on the left. (A) Phosphorylation of human MCM2-7 (50 nM) by Chk1 and Chk2. (B) Phosphorylation of mouse MCM2-7 (75 nM) by Chk1 and Chk2. (C) Phosphorylation of Drosophila and human MCM2-7 complexes (75 nM) by Drosophila Chk2 (300 nM). (D) Phosphorylation of Drosophila CMG and mouse MCM2-7 by Drosophila Chk2 (750 nM Chk2, 50 nM MCM2-7 or CMG).

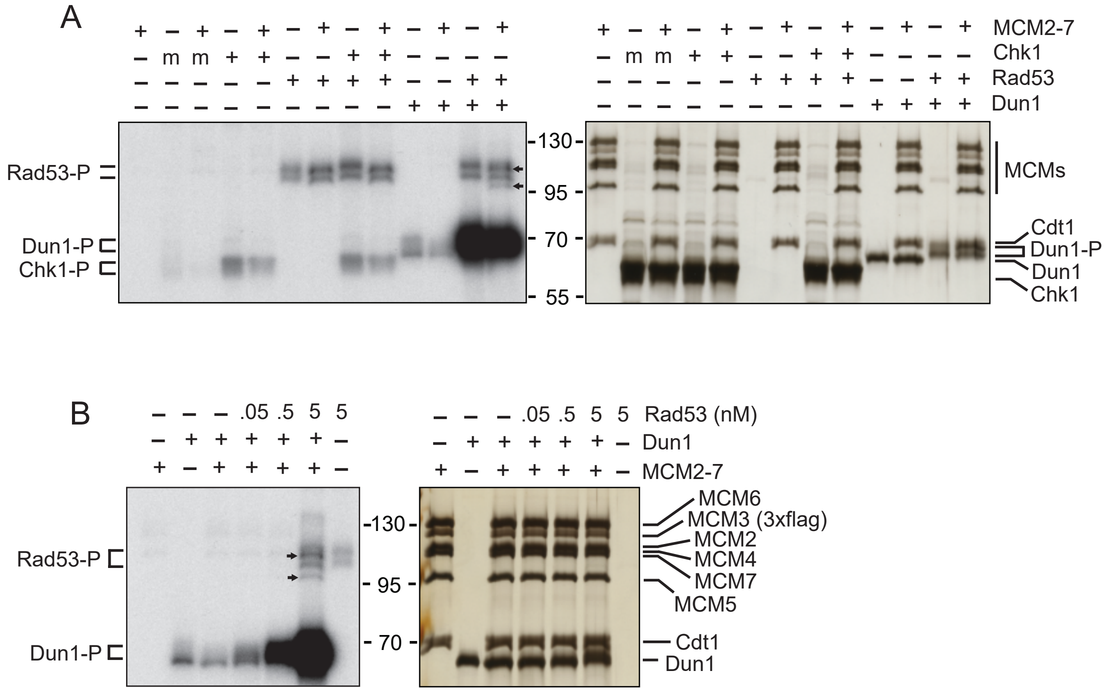

Figure S4. Kinase assays with budding yeast proteins. Silver-stained 10% SDS-PAGE protein gels are shown on the right and autoradiography film images of the same gels are on the left. (A) Phosphorylation of budding yeast MCM2-7 + Cdt1 (50 nM) by Chk1 (500 nM), Rad53 (10 nM), or Dun1 (50 nM) checkpoint kinases. 'm' indicates the lanes corresponding to the reactions with a kinase-defective Chk1 mutant (B) The Dun1-dependent phosphorylation of MCM subunits in response to the increasing concentrations of Rad53, which phosphorylates and activates the Dun1 kinase. The same concentrations of MCM2-7 + Cdt1 and Dun1 were used as in panel (A). Arrows in the autoradiographs indicate the Dun1-phosphorylated MCM subunit bands. The Rad53 levels used in these assays were below the sensitivity threshold of the silver staining protocol.

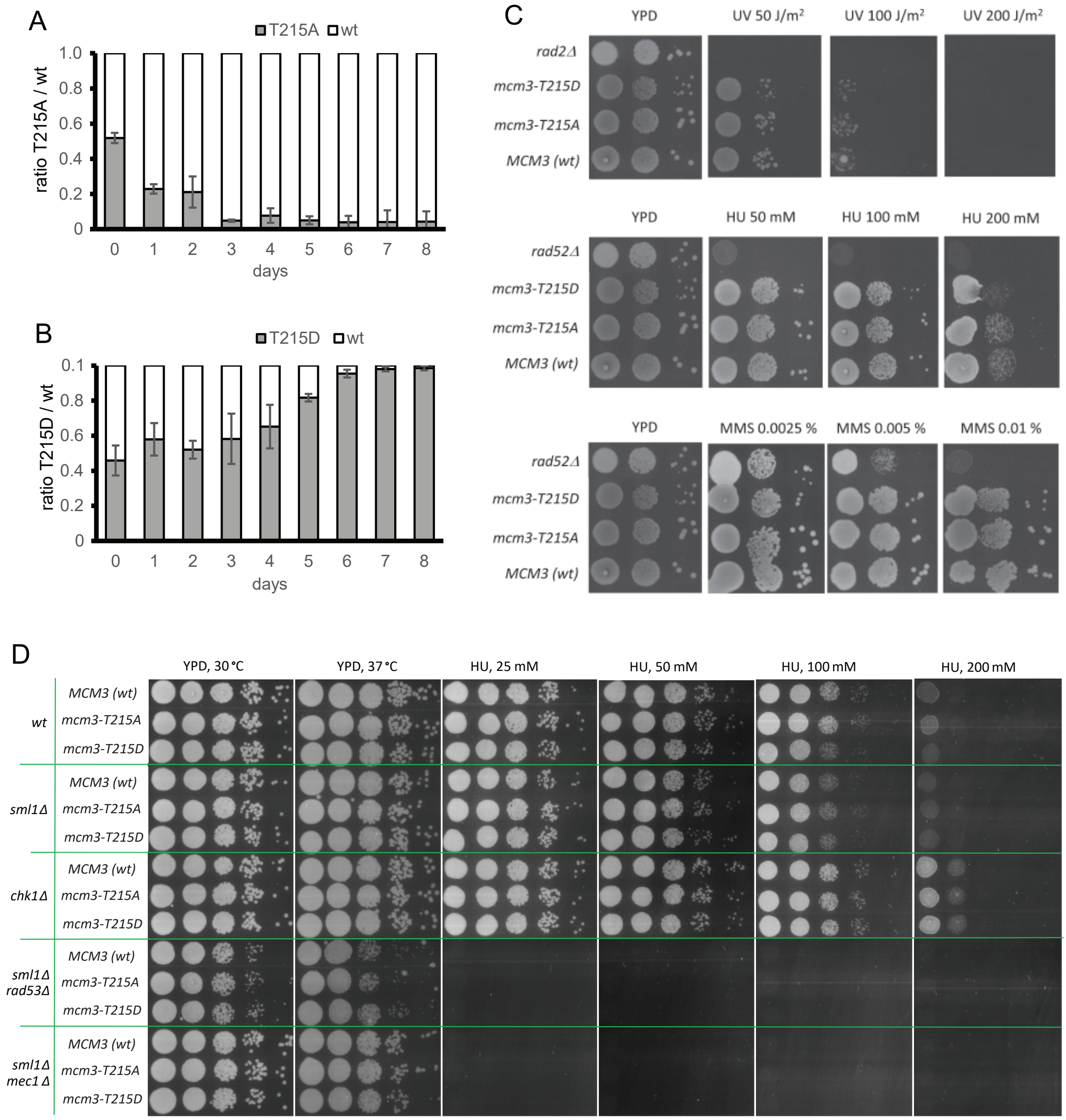

Figure S5. (A-B) Co-growth assays with wt budding yeast and mcm3-T215A (A) or mcm3-T215D (B) mutant strains. Graphs show the proportion of wt and mutant cells in the co-culture on the indicated day of growth, with the initial starting culture containing equal amounts of both. The average values from three experiments are presented, with error bars corresponding to the standard deviation. (C) A ten-fold dilution series of budding yeast *mcm3-T215A* or *T215D* mutant strains, together with the wild-type *MCM3* strain, were seeded on YPD plates. In the upper panel, the strains were treated with different doses of UV light and grown for three days at 30°C. In the middle and bottom panels, the strains were grown for three days on YPD plates at 30°C containing different concentrations of hydroxyurea or methyl methanesulfonate. The *rad2Δ* and *rad52Δ* strains served as controls because of their impaired capacity to respond to these treatments. (D) A ten-fold dilution series of wild-type *MCM3*, *mcm3-T215A*, or *mcm3-T215D* strains containing additional deletions of the indicated checkpoint kinase genes were seeded on YPD plates and grown for three days at 30°C, 37°C, or 30°C in the presence of different concentrations of hydroxyurea.

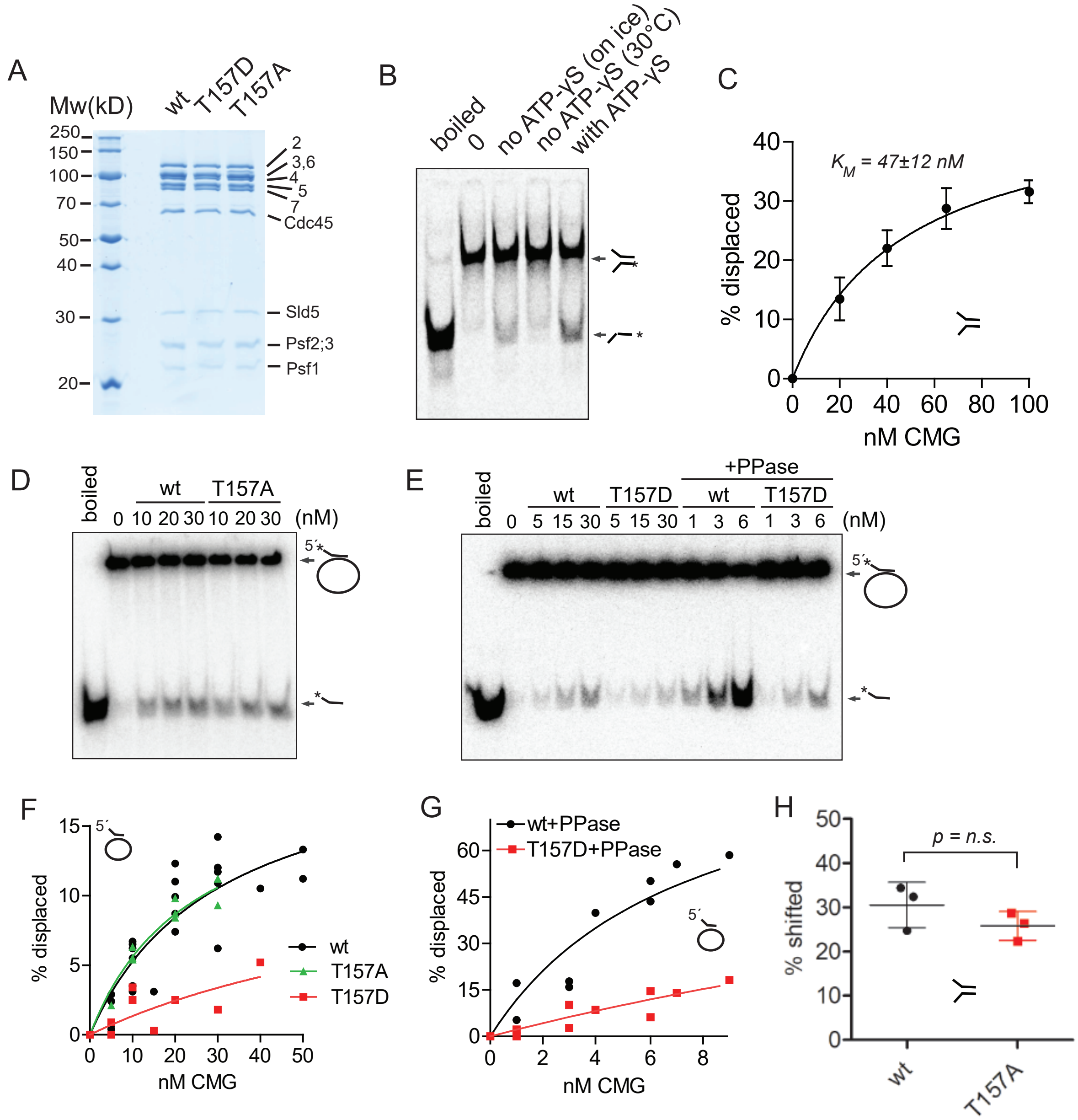

Figure S6. Helicase and EMSA assays with recombinant Drosophila CMG. (A) Coomassie brilliant blue-stained 10% SDS-PAGE gel with recombinant CMG proteins. (B) Helicase assay confirming the positive effect of pre-incubation with ATP-γS on the substrate unwinding. On all the autoradiography images of this figure, the arrows on the right mark the double-stranded substrate and displaced oligo bands; 'boiled' and '0' label the control lanes with the heat-denatured substrate or without protein, respectively. 50 nM wt CMG was pre-incubated either without (on ice or at 30°C as indicated) or with ATP-γS before adding ATP. (C) Michaelis-Menten kinetics curve of the helicase activity. Here, the indicated concentrations of wt CMG were pre-incubated with ATP-γS before ATP was added to initiate the unwinding. Data were collected from three replicate experiments and each point presents mean ± standard deviation. (D-G) Helicase assays conducted to compare the activities of the wild-type (wt) and specified mutant CMG complexes. The helicase activity was assessed using an M13-based probe and without prior incubation with ATP-γS in these experiments. '+PPase' indicates reactions with bacteriophage λ phosphatase pre-treated CMG. We performed eight independent experiments with phosphatase non-treated wt CMG and three with the other wt or mutant CMG proteins; panels (D-E) display representative autoradiographs from one assay. All quantified data points from these experiments, along with Michaelis-Menten kinetics curves based on this data, are presented in panels (F-G); note that CMG concentration points measured did not always overlap across experiments. (H) EMSA assay comparing the binding of wt or MCM3-T157A mutant CMG (120 nM) to the 1 nM of DNA fork. The data from three replicate experiments is shown together with median value and standard deviation. p values were calculated using the unpaired two-tailed t-test (n.s. – nonsignificant). Different forked substrate was used in these experiments compared to the EMSA assays in Fig. 6G-H (see Experimental procedures section for details).
